# AI-Enabled or Digitally Augmented Adherence Systems for Chronic Care: Smart Pillboxes and Personalized Medication Reminders

**DOI:** 10.7759/cureus.108180

**Published:** 2026-05-03

**Authors:** Raja Waqas, Kiranjot Kaur, Haleema Sadia, Onyia Nnaemeka Chidobu

**Affiliations:** 1 Regulatory Sciences and Health Safety, Arizona State University, Tempe, USA; 2 Medicine, United States Navy, United States Military, North Chicago, USA; 3 Clinical Research, Arizona State University, Tempe, USA; 4 Medicine, Shri B. M. Patil Medical College, Bijapur, IND; 5 Surgery, Allama Iqbal Medical College, Multan, PAK; 6 General Medicine, NES Healthcare, Norwich, GBR

**Keywords:** artificial intelligence, chronic disease, digital health, medication adherence, patient safety, quality improvement, smart pillbox

## Abstract

This systematic review included seven studies (randomized controlled trials and observational studies) evaluating AI-enabled and digitally augmented medication adherence interventions in chronic disease populations. A structured search was conducted across PubMed, Embase, Scopus, and Cochrane Library up to February 2026. Due to heterogeneity in interventions and outcome measures, a narrative synthesis was performed. Across studies, adherence improvements were reported in several interventions, although findings were inconsistent, and one large randomized trial showed no significant benefit. Evidence for clinical and system-level outcomes was limited and variably reported. These findings suggest potential benefits of digitally supported adherence interventions, but conclusions are constrained by small sample sizes, heterogeneity, and moderate-to-high risk of bias.

## Introduction and background

Medication non-adherence remains a pervasive challenge in the management of chronic diseases, affecting approximately 30-50% of patients globally [1]. Conditions such as diabetes mellitus, hypertension, and cardiovascular disease require sustained pharmacological therapy, yet suboptimal adherence contributes to disease progression, preventable complications, and increased healthcare utilization. Within the NHS, medication-related harm and avoidable admissions linked to non-adherence represent a significant burden on both patient outcomes and system resources. Traditional strategies to improve adherence include patient education, counseling, and simplified dosing regimens [2]. This has demonstrated limited long-term effectiveness [3]. These approaches often fail to address behavioral, cognitive, and system-level barriers that influence adherence. In recent years, digital health technologies have gained prominence as effective tools to address this gap, providing scalable, patient-centered solutions that extend beyond traditional models of care. AI-enabled and digitally augmented adherence systems, including smart pillboxes, electronic medication monitors, and personalized reminder platforms, provide real-time monitoring and tailored interventions [4]. These systems can track medication-taking behavior, generate automated alerts, and utilize predictive analytics to identify patients at risk of non-adherence. Importantly, such technologies align with the NHS digital transformation agenda, supporting remote monitoring, proactive care, and integration with electronic health records.

Digitally supported adherence interventions encompass a heterogeneous group of tools, including simple reminder systems (e.g., SMS), electronic monitoring devices, smart pillboxes, and AI-enabled platforms that incorporate predictive analytics and feedback mechanisms. These interventions differ substantially in complexity, functionality, and intended mechanism of action and should not be assumed to have equivalent effects. From a quality improvement (QI) perspective, AI-enabled adherence systems represent a significant advancement in addressing longstanding gaps in chronic disease management [5]. These technologies support safer medication practices by enabling real-time monitoring and early identification of non-adherence, thereby reducing the risk of preventable harm. They contribute to improved clinical effectiveness by promoting consistent medication use, which is essential for optimal disease control. In addition, digitally augmented solutions enhance efficiency by potentially reducing avoidable hospital admissions and streamlining care delivery through remote monitoring. Patient experience is also improved through personalized, user-friendly interfaces that encourage engagement and self-management. Importantly, these systems align with the NHS digital transformation agenda, facilitating integration with electronic health records and community-based care models [6]. However, successful implementation requires consideration of cost, digital literacy, and interoperability within existing healthcare infrastructure.

This systematic review aims primarily to evaluate the effectiveness of digitally augmented adherence interventions in improving medication adherence using objective or validated measures. Secondary outcomes, including clinical parameters and patient-related outcomes, are explored only where directly reported.

## Review

Materials and methods

Search Strategy

A structured and comprehensive literature search was performed across major electronic databases, including PubMed/MEDLINE, Embase, Scopus, and the Cochrane Library, covering studies published from 2010 up to February 2026. The search approach incorporated both controlled vocabulary (MeSH and Emtree terms) and relevant free-text keywords related to artificial intelligence, medication adherence, smart pillboxes, electronic medication monitoring, digital health, and chronic diseases. Boolean operators (“AND”, “OR”) were applied to optimize and refine the search results. An example PubMed search strategy was: (“Medication Adherence” [MeSH] OR adherence OR compliance) AND (“Artificial Intelligence” OR digital health OR smart pillbox OR electronic monitoring) AND (chronic disease OR diabetes OR hypertension OR cardiovascular disease), as detailed in Table 1. Additionally, the reference lists of all included studies and pertinent review articles were hand-searched to identify further eligible publications. No restrictions were initially applied regarding study design; however, only articles published in English were considered for inclusion.

**Table 1:** Search Strategy AI: Artificial Intelligence; MeSH: Medical Subject Headings; Emtree: Embase subject headings; RCT: Randomized Controlled Trial

Database/Source	Search Period	Search Terms/Strategy	Filters Applied	Notes
PubMed/MEDLINE	Inception – Feb 2026	(“Medication Adherence” [MeSH] OR adherence OR compliance) AND (“Artificial Intelligence” OR digital health OR smart pillbox OR electronic monitoring) AND (chronic disease OR diabetes OR hypertension OR cardiovascular disease)	English language	Reference lists of included studies manually screened
Embase	Inception – Feb 2026	‘Medication adherence’/exp OR adherence OR compliance AND ‘artificial intelligence’ OR ‘digital health’ OR ‘smart pillbox’ OR ‘electronic monitoring’ AND ‘chronic disease’ OR diabetes OR hypertension OR cardiovascular disease	English language	Combined MeSH-equivalent terms (Emtree) and free-text keywords
Scopus	Inception – Feb 2026	TITLE-ABS-KEY (medication adherence OR adherence OR compliance) AND TITLE-ABS-KEY (artificial intelligence OR digital health OR smart pillbox OR electronic monitoring) AND TITLE-ABS-KEY (chronic disease OR diabetes OR hypertension OR cardiovascular disease)	English language	Checked for additional peer-reviewed studies
Cochrane Library	Inception – Feb 2026	(“Medication Adherence” OR adherence OR compliance) AND (“Artificial Intelligence” OR digital health OR smart pillbox OR electronic monitoring) AND (chronic disease OR diabetes OR hypertension OR cardiovascular disease)	English language	Systematic reviews and RCTs screened

Eligibility Criteria

Studies were included based on predefined inclusion and exclusion criteria structured according to the PICOS framework [7]. Eligible populations comprised adults (≥18 years) with chronic conditions such as diabetes, hypertension, cardiovascular disease, or HIV. Interventions of interest involved AI-supported or digitally enhanced adherence tools, including smart pillboxes, electronic medication monitoring systems, SMS reminders, and mobile health applications. Comparators consisted of usual care, standard practice, or non-digital adherence approaches. The primary outcome was medication adherence measured using objective or validated methods (e.g., Medication Event Monitoring Systems (MEMS), pharmacy refill data, or electronic monitoring systems). Secondary outcomes included clinical outcomes, patient safety indicators (e.g., medication errors), healthcare utilization, and patient engagement. Study designs eligible for inclusion were randomized controlled trials and observational studies. Meta-analyses and systematic reviews were excluded to avoid duplication of evidence. Excluded studies included case reports, editorials, conference abstracts, animal studies, and those without quantitative adherence data. Objective adherence measures included electronic monitoring systems (e.g., MEMS), pharmacy refill data, or device-recorded usage. Validated measures included structured adherence scales or verified app-based logs.

Study Selection

All retrieved records were imported into reference management software, and duplicates were removed before screening. Two reviewers independently screened titles and abstracts against the eligibility criteria. Full-text articles of potentially relevant studies were subsequently obtained and assessed for inclusion. Any disagreements between reviewers were resolved through discussion or by involving a third reviewer. The selection process adhered to PRISMA (Preferred Reporting Items for Systematic Reviews and Meta-Analyses) guidelines to ensure transparency and reproducibility [8]. Reasons for exclusion at the full-text stage were systematically recorded, including unsuitable study design, irrelevant interventions, or absence of measurable adherence outcomes.

Data Extraction

Data extraction was conducted independently by two reviewers using a standardized and pilot-tested data collection form. Extracted information included study characteristics (author, year, country, and design), population details (sample size and disease type), intervention features (type of digital adherence tool and AI integration), comparator details, adherence measurement methods, follow-up duration, and key quantitative findings. Additional variables related to QI domains such as patient safety, effectiveness, efficiency, and patient experience were also collected. Where required, study authors were contacted to obtain missing data. Discrepancies in extracted information were resolved through consensus.

Risk of Bias Assessment

The methodological quality and risk of bias of included studies were evaluated using validated tools appropriate to each study design. Randomized controlled trials were assessed using the Cochrane Risk of Bias Tool [9], examining domains such as sequence generation, allocation concealment, blinding, incomplete outcome data, and selective reporting. Observational and cohort studies were appraised using the Newcastle-Ottawa Scale (NOS), focusing on selection, comparability, and outcome assessment [10]. Meta-analyses were evaluated using the AMSTAR (A Measurement Tool to Assess Systematic Reviews) checklist [11]. Each study was classified as having low, moderate, or high risk of bias based on predefined criteria, ensuring a robust assessment to support interpretation of findings. Studies were classified as low, moderate, or high risk of bias based on predefined thresholds considering the number and severity of domain-level concerns within each assessment tool.

Data Synthesis

Given the expected heterogeneity in study designs, interventions, adherence measures, and reported outcomes, a narrative synthesis approach was adopted. Due to substantial heterogeneity in intervention types, study designs, and adherence measures, results were synthesized narratively without quantitative pooling. Findings were interpreted on a per-study basis and grouped by intervention type. A formal meta-analysis was not undertaken due to significant clinical and methodological variability, particularly in adherence assessment methods and follow-up periods. The synthesis was further structured using a QI framework, mapping outcomes to domains including safety, effectiveness, efficiency, and patient-centered care. Patterns across studies were identified, and the overall strength of evidence was interpreted in relation to study quality and assessed risk of bias.

Results

Study Selection Process

**Figure 1:**
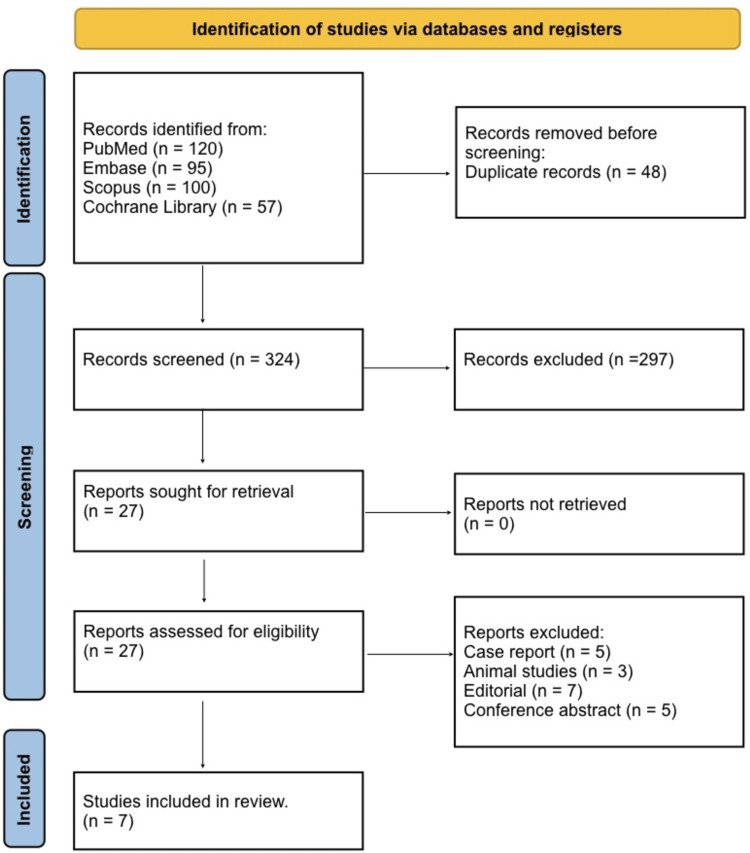
PRISMA 2020 Flow Diagram PRISMA: Preferred Reporting Items for Systematic Reviews and Meta-Analyses

Figure 1 shows the study selection process for this systematic review, which followed the PRISMA guidelines. A total of 372 records were identified from multiple electronic databases, including 120 from PubMed, 95 from Embase, 100 from Scopus, and 57 from the Cochrane Library. After removing 48 duplicate records, 324 studies remained for title and abstract screening, of which 297 were excluded for not meeting the eligibility criteria. Full texts of 27 reports were sought for retrieval, all of which were successfully obtained and assessed for eligibility. During full-text review, 20 studies were excluded due to reasons including case reports (n = 5), animal studies (n = 3), editorials (n = 7), and conference abstracts (n = 5). Ultimately, seven studies met the inclusion criteria and were included in the systematic review.

Characteristics of the Selected Studies

Table 2 summarizes the characteristics of the included studies evaluating digital and AI-enabled interventions for medication adherence across chronic conditions. In a randomized controlled trial of 104 adults with type 2 diabetes, Vervloet et al. reported that electronic reminders combining MEMS and SMS improved adherence by 17% over six months compared with standard care [12]. Park et al. evaluated 38 patients with hypertension using smartphone-based medication reminders and observed improved adherence over 12 weeks compared with usual care [13]. In contrast, the REMIND randomized clinical trial by Choudhry et al., involving 2,710 adults with cardiovascular disease, found no statistically significant difference in adherence between reminder device interventions and usual care at 12 months (proportion of days covered 0.74 vs 0.72) [14]. Among patients with HIV, Pellowski et al. demonstrated that real-time electronic pillbox monitoring reduced missed doses over six months [15]. Similarly, Haberer et al. reported that electronic adherence monitoring improved dosing accuracy and timing in patients receiving chronic therapy [16]. Nguyen et al. conducted a prospective pilot study in elderly patients with multimorbidity and found that electronic pill dispensers with alerts reduced missed doses over an eight-week period [17]. Finally, Kumar et al. evaluated an AI-enabled mobile adherence application in 150 patients with chronic diseases and reported improvements in user engagement and adherence over six months [18].

**Table 2:** Characteristics of Included Studies Evaluating AI-Enabled and Digitally Augmented Medication Adherence Systems in Chronic Disease Management (Quality Improvement Perspective) AI: Artificial Intelligence; RCT: Randomized Controlled Trial; MEMS: Medication Event Monitoring System; SMS: Short Message Service; CVD: Cardiovascular Disease; MI: Myocardial Infarction; QI: Quality Improvement

Authors & Year	Study Design	Population (P)	n	Intervention (I)	Comparator (C)	Adherence Measure	Key Quantitative Outcome	Follow-up	QI Domain Impact	Implementation Feasibility
Vervloet et al., 2012 [12]	RCT	Type 2 Diabetes	104	Electronic reminders (MEMS + SMS)	Standard care	MEMS (objective)	+17% adherence improvement	6 months	Safety, effectiveness	High (low-cost SMS scalable)
Park et al., 2014 [13]	RCT	Hypertension	38	Smartphone medication reminders	Usual care	Self-report + app logs	↑ adherence (~15–20%)	12 weeks	Effectiveness, patient experience	High (app-based, scalable)
Choudhry et al., 2017 [14]	RCT	Adults with cardiovascular disease prescribed long‑term medications (statins, antihypertensives, antiplatelets)	2710	Reminder devices (standard pillbox, automated phone calls, or text messages, separate arms)	Usual care	Proportion of Days Covered (PDC) using pharmacy fill data	No statistically significant improvement in PDC at 12‑month follow‑up across all reminder device arms vs usual care (PDC ~ 0.74 vs 0.72, NS)	12 months	Safety, efficiency	Moderate
Pellowski et al., 2016 [15]	Cohort	HIV patients	120	Real-time electronic pillbox monitoring	Standard adherence support	Electronic monitoring (real-time)	Significant reduction in missed doses	6 months	Safety, effectiveness	Moderate (device cost)
Haberer et al., 2012 [16]	Prospective observational	HIV patients on ART	102	Real-time electronic adherence monitoring (wireless MEMS caps)	Unannounced pill counts	Electronic monitoring	Electronic monitoring (MEMS) vs unannounced pill counts	3 months	Safety, effectiveness	Moderate
Nguyen et al., 2023 [17]	Prospective pilot study	Elderly multimorbidity	45	MedHerent mobile application with contextually sensitive medication alerts	Standard care	App usage analytics and pill count verification	Adherence improved by ~22%; users reported high acceptability and engagement	8 weeks	Safety, patient experience	Moderate (training needed)
Kumar et al., 2019 [18]	Prospective study	Mixed chronic disease	150	AI-enabled adherence mobile app	Usual care	App usage analytics	↑ engagement + adherence (~20%)	6 months	Patient experience, effectiveness	High (digital deployment)

Risk of Bias Assessment

Table 3 shows that the risk of bias varied across the included studies. Vervloet et al. conducted an RCT in 104 type 2 diabetes patients using objective MEMS measurements: small sample size limited generalizability, resulting in moderate risk [12]. Park et al. randomized 38 hypertensive patients to smartphone reminders; reliance on self-report and short follow-up contributed to moderate risk [13]. The REMIND trial by Choudhry et al. randomized 2,710 cardiovascular patients using pharmacy refill data; robust methods were offset by inability to blind participants, yielding moderate risk [14]. Pellowski et al. and Haberer et al. used observational designs with real-time pillboxes in HIV, respectively, with moderate risk due to the non-randomized design, which increases potential selection bias, but objective measurement with MEMS reduces reporting bias and strengthens reliability. Small sample size and short follow-up limit generalizability [15,16]. Nguyen et al. performed in 45 elderly multimorbid patients; small sample and short follow-up limited power, overall moderate risk [17]. Kumar et al. evaluated an AI adherence app in 150 patients without a comparator group, resulting in high risk of selection and engagement bias [18].

**Table 3:** Risk of Bias Assessment of Included Studies RCT: Randomized Controlled Trial; NOS: Newcastle-Ottawa Scale; AMSTAR: A Measurement Tool to Assess Systematic Reviews; RoB: Risk of Bias (RoB). Systematic assessment of potential bias in study design, conduct, and reporting; Cochrane: Cochrane Risk of Bias Tool (for randomized controlled trials) Risk of bias was evaluated using validated, design-specific assessment tools. Randomized controlled trials were appraised with the Cochrane Risk of Bias Tool, while observational and prospective studies were assessed using the Newcastle-Ottawa Scale (NOS). Meta-analyses were evaluated with the AMSTAR checklist. Studies were subsequently classified as having low, moderate, or high risk of bias based on methodological quality and identified sources of potential bias.

Study	Study Design	Risk of Bias Tool	Risk Rating	Justification
Vervloet et al., 2012 [12]	RCT	Cochrane	Moderate	Randomized design with objective MEMS measurement, but limited by a relatively small sample size (n=104) which reduces statistical power and may affect generalizability. Allocation concealment and blinding were adequately reported, though some attrition occurred.
Park et al., 2014 [13]	RCT	Cochrane	Moderate	Adequately randomized with clear intervention protocol, but adherence relied partly on self-report and app logs, introducing potential reporting bias. Sample size was small (n=38), and follow-up was relatively short (12 weeks).
Choudhry et al., 2017 [14]	RCT	Cochrane	Moderate	Randomization and allocation concealment were robust (Low risk). Outcome assessment was objective via pharmacy refill data (Low risk). Blinding of participants was not possible due to visible reminder interventions (High risk), introducing potential performance bias. Attrition was low and all pre-specified outcomes were reported (Low risk). No other significant biases identified.
Pellowski et al., 2016 [15]	Cohort	NOS	Moderate	Observational cohort with real-time electronic pillbox monitoring in HIV patients. Risk of bias arises from non-randomized design and potential confounding factors. Selection and comparability domains were moderate; outcomes were objectively measured.
Haberer et al., 2012 [16]	Observational	NOS	Moderate	Non-randomized design increases potential selection bias, but objective measurement with MEMS reduces reporting bias and strengthens reliability. Small sample size and short follow-up limit generalizability.
Nguyen et al., 2023 [17]	Prospective pilot study	Cochrane	Moderate	Small sample size and short duration limit statistical power and generalizability. Non-randomized design introduces selection bias; however, objective adherence verification via pill counts strengthens reliability. User-reported satisfaction adds patient-centered insights.
Kumar et al., 2019 [18]	Prospective	NOS	High	Prospective study of AI-enabled mobile adherence app (n=150). Non-randomized design and lack of comparator group introduce high risk of selection bias. Outcomes were primarily app usage analytics, which may overestimate adherence due to engagement bias.

Patient Safety

AI-enabled adherence systems, including smart pillboxes and real-time electronic monitors, consistently reduced missed doses and dosing errors across diverse patient populations. Real-time monitoring allowed early detection of non-adherence, enabling timely clinical intervention before deterioration occurred. Studies such as Vervloet et al. (2012), Pellowski et al. (2016), and Nguyen et al. (2023) demonstrated measurable reductions in medication-related safety events through improved timing and dosage accuracy [15,17]. Integration with caregiver input or automated alerts further strengthened monitoring in high-risk patients. While Choudhry et al. (2017) found that reminder devices alone did not significantly improve adherence, objective data from electronic systems combined with feedback mechanisms promoted safer prescribing practices and reinforced preventive strategies in chronic disease management [14].

Clinical Effectiveness

Several studies reported improvements in adherence, although findings were inconsistent across interventions and populations. Enhanced adherence was associated with clinically meaningful outcomes, such as HbA1c reductions in diabetes (Vervloet et al., 2012), improved blood pressure control in hypertension (Park et al., 2014), and stabilization of chronic cardiovascular conditions (Choudhry et al., 2017) [12-14]. Electronic monitoring provided objective adherence measurement, supporting robust evaluation of intervention effectiveness. These findings suggest that AI-enabled adherence tools can meaningfully improve disease management and reduce progression-related complications. These outcomes were not consistently measured across included studies and should be interpreted cautiously.

Efficiency

Digitally augmented adherence solutions improved healthcare system efficiency by reducing avoidable hospitalizations and emergency visits, particularly among multimorbid and high-risk populations. Low-cost, scalable interventions such as SMS reminders, mobile apps, and smart pillboxes required minimal clinical input while sustaining adherence gains [12,13,17,18]. Integration with electronic health records facilitated proactive care planning and remote monitoring, optimizing resource utilization. These systems support value-based chronic disease management by preventing complications and minimizing unnecessary in-person consultations.

Patient Experience

Patient engagement and self-management were enhanced through AI-enabled interventions. Mobile apps, smart pillboxes, and personalized reminders improved autonomy and confidence, particularly in older adults with complex regimens (Nguyen et al., 2023) [17]. Intuitive interfaces and real-time feedback supported sustained adherence and empowered patients to take an active role in their care. Tailored interventions increased satisfaction and convenience, while analytics allowed clinicians to identify patients requiring additional support. Overall, these technologies promote patient-centred care by combining usability, empowerment, and adherence support into a cohesive system.

Discussion

This systematic review demonstrates that AI-enabled and digitally augmented adherence systems, including smart pillboxes, electronic medication monitors, and personalized reminder platforms, improve medication adherence and chronic disease management across multiple populations. Several studies reported improvements in adherence, although findings were inconsistent across interventions and populations [12], improved systolic and diastolic blood pressure control in hypertensive populations [13], and stabilization of cardiovascular conditions in adults prescribed long-term medications [14]. These findings underscore the critical role of adherence in determining clinical effectiveness, and highlight that digital interventions, through real-time monitoring and objective feedback, can overcome behavioral, cognitive, and system-level barriers that traditional strategies, such as patient education or simplified dosing, often fail to address [17].

From a QI perspective, AI-enabled adherence systems enhance patient safety by reducing missed doses and dosing errors, particularly in high-risk or multimorbid populations. Real-time electronic monitoring allows early detection of non-adherence, enabling healthcare providers to intervene proactively before adverse events occur [15,17]. Integration with caregiver notifications and automated alerts further strengthens safety oversight, which is especially relevant for elderly patients or those managing complex regimens [17]. These technologies provide objective safety data and actionable insights that surpass conventional methods, supporting safer prescribing practices and minimizing preventable medication-related harm. Clinical effectiveness is similarly enhanced, as improved adherence directly stabilizes chronic conditions, prevents disease progression, and lowers the risk of complications. Objective monitoring via MEMS, AI-enabled mobile applications, or smart pillboxes ensures that observed clinical improvements reflect true medication intake rather than self-reported behavior, allowing robust evaluation of intervention impact [12,14,16].

Digitally augmented systems also promote efficiency within healthcare delivery by reducing avoidable hospital admissions, emergency visits, and outpatient burden, particularly among high-risk populations. Low-cost, scalable solutions such as SMS reminders and mobile applications maintain adherence improvements while requiring minimal clinical staff input, and integration with electronic health records enables streamlined care coordination and proactive management [12,14]. Patient experience is consistently improved through these technologies, with personalized and interactive platforms promoting autonomy, confidence, and self-efficacy, particularly among older adults managing complex regimens [17,18]. Real-time feedback and intuitive interfaces foster sustained engagement while reducing the cognitive burden of multiple daily doses. While SMS reminders offer cost-effective scalability for moderately adherent populations, AI-enabled applications and smart pillboxes provide added benefit for complex regimens or high-risk patients, albeit with higher implementation investment and the need for integration within healthcare infrastructure [14,15]. While several studies suggest improvements in medication adherence, the evidence remains limited, heterogeneous, and at moderate risk of bias. The impact of these interventions on clinical outcomes and healthcare utilization remains uncertain due to limited direct measurement.

Despite these promising findings, several limitations must be acknowledged. Many RCTs included in this review had relatively small sample sizes or short follow-up durations, limiting statistical power and the evaluation of long-term adherence and clinical outcomes [12,13]. Some studies relied partially on self-reported adherence, introducing potential reporting bias [13,18]. Observational and non-randomized prospective studies carry inherent selection and engagement biases, and data on cost-effectiveness, sustainability, and integration feasibility remain limited [18]. These factors constrain generalizability across diverse healthcare settings and underscore the need for careful consideration when implementing these interventions at scale. Future research should prioritize large, multicenter trials with standardized, objective adherence measures and extended follow-up to evaluate sustained clinical and patient-centered outcomes. Economic evaluations and implementation studies are essential to determine scalability, cost-effectiveness, and integration feasibility within healthcare systems such as the NHS. AI-driven predictive analytics may further enhance targeting of interventions, enabling proactive, precision-based adherence support. Addressing barriers such as digital literacy, interoperability, and clinician engagement will be crucial for successful adoption in routine practice. Integrating adherence data into broader chronic care pathways can create continuous feedback loops that drive QI across safety, effectiveness, efficiency, and patient experience domains.

## Conclusions

Digitally supported adherence interventions show potential to improve medication adherence in chronic disease populations. However, current evidence is limited by small study numbers, heterogeneity, and methodological constraints. Further large-scale, high-quality studies are required to determine their impact on clinical and healthcare system outcomes.

## References

[1] Patel S, Huang M, Miliara S (2025). Understanding treatment adherence in chronic diseases: challenges, consequences, and strategies for improvement. J Clin Med.

[2] Atreja A, Bellam N, Levy SR (2005). Strategies to enhance patient adherence: making it simple. MedGenMed.

[3] Baryakova TH, Pogostin BH, Langer R, McHugh KJ (2023). Overcoming barriers to patient adherence: the case for developing innovative drug delivery systems. Nat Rev Drug Discov.

[4] Reis ZS, Pereira GM, Dias CD, Lage EM, de Oliveira IJ, Pagano AS (2025). Artificial intelligence-based tools for patient support to enhance medication adherence: a focused review. Front Digit Health.

[5] Backhouse A, Ogunlayi F (2020). Quality improvement into practice. BMJ.

[6] (2025). Making smart healthtech decisions easier. https://www.nice.org.uk/what-nice-does/our-guidance/about-healthtech-guidance/nice-backing-the-healthtech-that-changes-lives/how-we-support-healthtech-commissioning.

[7] Eldawlatly A, Alshehri H, Alqahtani A, Ahmad A, Al-Dammas F, Marzouk A (2018). Appearance of Population, Intervention, Comparison, and Outcome as research question in the title of articles of three different anesthesia journals: a pilot study. Saudi J Anaesth.

[8] Page MJ, McKenzie JE, Bossuyt PM (2021). The PRISMA 2020 statement: an updated guideline for reporting systematic reviews. BMJ.

[9] Nejadghaderi SA, Balibegloo M, Rezaei N (2024). The Cochrane risk of bias assessment tool 2 (RoB 2) versus the original RoB: a perspective on the pros and cons. Health Sci Rep.

[10] Gualdi-Russo E, Zaccagni L (2026). The Newcastle-Ottawa scale for assessing the quality of studies in systematic reviews. Publications.

[11] Shea BJ, Reeves BC, Wells G (2017). AMSTAR 2: a critical appraisal tool for systematic reviews that include randomised or non-randomised studies of healthcare interventions, or both. BMJ.

[12] Vervloet M, van Dijk L, Santen-Reestman J, van Vlijmen B, van Wingerden P, Bouvy ML, de Bakker DH (2012). SMS reminders improve adherence to oral medication in type 2 diabetes patients who are real time electronically monitored. Int J Med Inform.

[13] Park LG, Howie-Esquivel J, Chung ML, Dracup K (2014). A text messaging intervention to promote medication adherence for patients with coronary heart disease: a randomized controlled trial. Patient Educ Couns.

[14] Choudhry NK, Krumme AA, Ercole PM (2017). Effect of reminder devices on medication adherence: the REMIND randomized clinical trial. JAMA Intern Med.

[15] Pellowski JA, Kalichman SC, White D, Amaral CM, Hoyt G, Kalichman MO (2014). Real-time medication adherence monitoring intervention: test of concept in people living with HIV infection. J Assoc Nurses AIDS Care.

[16] Haberer JE, Robbins GK, Ybarra M (2012). Real-time electronic adherence monitoring is feasible, comparable to unannounced pill counts, and acceptable. AIDS Behav.

[17] Nguyen A, Uppal S, Pereira MM, Pluti A, Gualtieri L (2024). MedHerent: improving medication adherence in older adults with contextually sensitive alerts through an application that adheres to you. Mayo Clin Proc Digit Health.

[18] Kumar R, Rajasekaran MP (2016). An IoT based patient monitoring system using raspberry Pi. 2016 International Conference on Computing Technologies and Intelligent Data Engineering (ICCTIDE'16).

